# Comparison of Fractionated Dose Versus Bolus Dose of Hyperbaric Bupivacaine Injection in Spinal Anesthesia: Hemodynamic Stability in Elderly Patients Undergoing Lower Limb Surgeries

**DOI:** 10.7759/cureus.88714

**Published:** 2025-07-25

**Authors:** Shailesh Mishra, Neha Dubey, Akash Verma, Shalini Sahu

**Affiliations:** 1 Department of Anaesthesiology, Peoples College of Medical Sciences and Research Centre, Bhopal, IND; 2 Department of Anaesthesiology, Atal Bihari Vajpayee Government Medical College, Vidisha, IND

**Keywords:** anesthesia, bolus dose, fractionated dose, spinal anesthesia, spinal anesthesia bolus dose

## Abstract

Background: Spinal anesthesia (SA) with a bolus dose provides a rapid onset but often leads to hemodynamic instability, particularly in elderly patients. Fractionated dosing of hyperbaric bupivacaine may offer a dense block with greater stability and prolonged analgesia. This study compares the effects of bolus and fractionated dosing on hemodynamic parameters in elderly patients undergoing lower limb surgeries.

Methods: In this randomized controlled trial, 60 elderly patients (n=60 patients; 30 patients per group) of more than 60 years were randomly assigned to receive either a bolus dose (Group B) or fractionated doses (Group F) of hyperbaric bupivacaine for SA. Intraoperative monitoring included blood pressure, heart rate, oxygen saturation, and ECG. Hemodynamic parameters were analyzed at serial intervals up to 180 minutes.

Results: Demographic and baseline parameters were comparable between groups. A statistically significant difference in mean arterial pressure (MAP) and diastolic blood pressure (DBP) was observed only at 90 minutes post-anesthesia (MAP: Group F 90.71 mmHg vs. Group B 84.63 mmHg, p = 0.043; DBP: Group F 74.90 mmHg vs. Group B 69.04 mmHg, p = 0.025). The fractionated group required fewer vasopressor interventions (p = 0.001).

Conclusion: Fractionated dosing of hyperbaric bupivacaine in SA demonstrated improved hemodynamic stability at specific time points and reduced vasopressor requirements in elderly patients. However, due to limited power and isolated significance, further large-scale, blinded studies are needed to confirm the clinical relevance of fractionated dosing in elderly patients.

## Introduction

Spinal anesthesia (SA) is one of the most common anesthetic techniques in which a local anesthetic drug is injected into the subarachnoid space. The drug is delivered using a special kind of needle called a spinal needle. The injection is usually made in the L2/3 or L3/4 intervertebral space [1]. This form of neuraxial technique has been successfully used in children, adults, and geriatric patients. Due to better medical facilities, the geriatric population is gradually increasing in India. Incidence of co-existing conditions is high in the geriatric age group, so selection of the optimal mode of anesthesia is very important, as any particular type of anesthesia is not universally acceptable. Thus, anesthetic technique should be carefully individualized for elderly patients [2]. The onset of action of subarachnoid blockade is fast, and it has no significant effect on mental functions. Moreover, less intraoperative loss of blood volume is also seen with it. Such properties make SA “The anesthesia of choice” for surgical procedures of the lower limb [3].

SA commonly causes hypotension via sympathetic blockade and decreased cardiac output due to a reduction in preload secondary to sympatholysis. Geriatric patients have poor physiological reserves; thus, they show a greater degree of hypotension, resulting in decreased perfusion to various vital organs. Hence, the drug dosage of subarachnoid blockade is routinely revised, and a lower dose is applied to patients of this age group [4].

The concept of low-dose SA is very useful in patients who are more than 60 years of age. There are different methods of giving low dosages, like continuous SA, fractionated dosing, etc. In the fractionated dosage method, the total calculated dose of local anesthetic is divided into two portions and given one after the other with a small time gap, like 60 or 90 seconds. In this method, the patients show lower incidences of fall in blood pressure, and sensory and motor blockade persisted for a longer time interval [5]. Patients remained pain-free for longer time intervals in post-surgical units.

Another concept of the total dose of local anesthetic drug is to apply the drug with adjuvants like opioids, thus attenuating its hypotensive side effects. In continuous SA (CSA), too, there is the advantage of giving a titrated low dose of local anesthetic through a catheter into the subarachnoid space, which is known to show a better hemodynamic profile [2]. A major limitation of bolus dosing is the limited duration of anesthesia. With unanticipated prolongation of surgery, the duration of anesthesia cannot be increased. This limitation can be dealt with effectively by using continuous SA, in which anesthesia duration can be increased by repeating intrathecal administration of local anesthetic [6,7].

When SA is given in small fractions, fewer fluctuations in vital parameters are seen, and the evolution of sensory block can be assessed before giving the total calculated dose. In case of an emergency surgery, early evolution of sensory block facilitates early incision and start of surgery before attaining maximal blockade height [8].

Bupivacaine is a local anesthetic that comes under the amide group. It is used very frequently in 0.25%w/v and 0.5%w/v strengths for producing anesthesia. It may be used for local infiltration, blocking peripheral nerves, or in neuraxial anesthesia like epidural or subarachnoid blocks. It is a long-acting pipecoloxylidide and is used in conditions where prolonged anesthetic action is required. As compared to motor block, its sensory blocking effect is more marked, which gives prolonged analgesic duration in the postoperative period [9,10]

Levobupivacaine is an S isomer of bupivacaine, which has grossly similar pharmacokinetic properties to bupivacaine. Bupivacaine is associated with side effects like cardiac and neurological alterations, but these effects are less pronounced with levobupivacaine. Nowadays, levobupivacaine is being used frequently in regional anesthesia administration [6].

Fractionated doses of SA are known for lesser fluctuations in intraoperative vital parameters along with longer durations of postoperative analgesia as compared to single-dose SA (SDSA) [11].

The current study is to compare the intraoperative hemodynamic stability in a fraction and bolus dose of injectable bupivacaine heavy 0.5% in a subarachnoid block in elderly patients undergoing lower limb surgeries. The primary objective was to compare mean arterial pressure (MAP) trends over time between the two techniques, while the secondary objective was to assess the incidence of hypotension, bradycardia, and vasopressor requirement.

## Materials and methods

This prospective randomized controlled trial was conducted over a three-month period, from October 1, 2017, to December 31, 2017. The preparatory phase, including protocol development and logistical arrangements, commenced on October 1, 2017. Ethical approval for the study was granted on October 26, 2017. After obtaining approval from the Institutional Ethics Committee, adhere to ethical standards throughout the research process at People's College of Medical Sciences and Research Centre, Bhopal. No data collection or participant interaction occurred prior to obtaining ethical approval. Data collection commenced on October 27, 2017, and continued through December 31, 2017.

A sample size of 60 elderly patients (age more than 60 years) posted for lower limb surgeries with a plan of SA was included.

After the Institutional Ethics Committee approval and written informed consent, the present study was carried out on 60 elderly (age more than 60 years) patients. Calculation of sample size- The sample size was calculated based on the expected difference between the two groups (effect size), the standard deviation from previous studies, a significance level (α) of 0.05, and a power of 80% (β = 0.2).

n= [(Z_α/2_+Zβ/δ/σ)^2^] x2

Where:

Z_α/2_ = 1.96 for α = 0.05 (two-tailed), Zβ = 0.84 for 80% power

δ = expected difference in means (effect size)

σ = pooled standard deviation

The ideal sample size was calculated as 88 based on standard assumptions. However, the study was conducted with 60 participants, which may limit its power to detect smaller effect sizes. A number of methodological, ethical, and practical factors led to this choice. The sample size of 30 participants per group (60 total) represents a pragmatic and ethical choice given the study’s 3-month duration, resource constraints, and the expected magnitude of effects.

The criteria for participant selection were carefully established. Inclusion criteria encompassed patients who had undergone lower limb surgeries and had American Society of Anesthesiologists (ASA) physical status 1 and 2 and were aged more than 60 years. Conversely, exclusion criteria included patients with ASA physical status 3 and 4, pre-existing cardiovascular or cerebrovascular disease, allergies or hypersensitivity to local anesthetic drugs, spinal deformities, and patients unwilling to provide consent. No biological materials were required for the study, streamlining the logistical aspects and focusing primarily on procedural and statistical methodologies.

The patients were randomly divided into two groups. Randomization was performed using a computer-generated random number table. Allocation concealment was ensured using sequentially numbered, opaque, sealed envelopes to prevent selection bias. Group B patients received a single bolus dose of bupivacaine, while Group F patients received a fractionated dose of bupivacaine with two-thirds of the total calculated dose given initially, followed by a one-third dose after 90 sec. Both doses were given at a rate of 0.2 ml/sec. For 90 seconds, the syringe was kept attached to the needle, after which the remaining one-third dose was administered. Onset and regression of sensory and motor blockade were noted. A large-bore IV cannula was secured, and patients were premedicated with ranitidine 1 mg/kg and ondansetron 0.1 mg/kg IV. A multipara monitor was attached, and baseline vitals were noted down. Non-invasive blood pressure (NIBP), electrocardiogram (ECG), pulse oximeter (SpO2), and heart rate (HR) monitoring were done. Preloading was done with IV Ringer's lactate (RL), 10-15 ml/kg over 10 min. Intravenous fluid was administered based on ideal body weight to ensure consistent dosing across patients and to avoid fluid overload, particularly in overweight individuals. Skin infiltration with 3 ml of 2% lignocaine was done in the space where a skin prick for the administration of SA was planned. SA was given between the third and fourth or fourth and fifth intervertebral spaces with Quincke's needle (25G). After aspiration of cerebrospinal fluid, 0.5% bupivacaine heavy was injected according to respective groups B and F. The total dose of SA was calculated as 0.09 mg/cm of the height of the patient on the basis of studies by Harten et al., Liu et al., Sell et al. and Mahan et al. [12-15] A pilot study in five patients was done using a lower dose of 0.07 mg/cm, but the duration and level of blockade were not found satisfactory; thus, the study was done on dose of 0.09 mg/cm.

Monitoring and data collection

Hemodynamic parameters, including systolic blood pressure (SBP), diastolic blood pressure (DBP), MAP, and HR, were recorded at baseline (pre-spinal), every 5 minutes for the first 30 minutes, and then every 10 minutes until the end of surgery.

Primary Outcome

Difference in MAP trends over time between the two groups.

Secondary Outcomes

Incidence of hypotension (defined as >20% decrease from baseline MAP or SBP <90 mmHg), incidence of bradycardia (defined as HR <50 bpm).

Requirement for Vasopressor Support

Hypotension was managed with IV mephentermine 6 mg boluses, repeated as necessary. Bradycardia was treated with IV atropine 0.6 mg.

Blinding

This was a single-blind study; participants were blinded to group allocation, but the anesthesiologist administering the drug was not, due to the nature of the interventions.

Statistical analysis

All the data analyses were performed using SPSS version. 20 software. Frequency distribution and cross-tabulation were performed to prepare the tables. Qualitative data, such as age and maximum dermatome achieved, were analyzed statistically using the chi-square test. Quantitative data were presented as mean ± standard deviation (SD) and analyzed using the paired sample t-test and individual sample t-test. The level of significance was assessed at 5%.

## Results

The mean age of patients in Group B and Group F was 73.17 and 69.83 years, respectively, while the mean height of patients in Group B and Group F was 150.47cm and 151.23cm, respectively. Genders were equally distributed in both groups. Demographically, the age, gender, height, and weight of patients in both groups showed no significant differences.

A comparison of drug doses in both groups is shown in Table 1.

**Table 1:** Comparing drug dose (ml) F: fractionated dose; B: bolus dose

Drug dose (ml)	Group	N	Mean	Std. Deviation	P-value
	B	30	2.73	0.37	1.000
	F	30	2.73	0.37	
	Total	60	2.7367	0.36	

On comparing the number of responders and non-responders to the pinprick at 10 minutes, it was found that responses were similar in both groups, as revealed by an insignificant p-value (p=0.448). There was no response at 20 and 30 minutes in patients from both groups.

In Group B, there were 7 (23.3%) with ASA grade I and 23 (76.6%) patients with ASA grade II, whereas in Group F, there were 10 (33.3%) with ASA grade I and 20 (66.6%) patients with ASA grade II. Intraoperative HRs were measured in both groups, as shown in Table 2.

**Table 2:** Comparing intraoperative vitals (Heart Rate) F: fractionated dose, B: bolus dose. Heart rate was observed at intervals of 10 minutes, 20 minutes, 30 minutes, 60 minutes, 90 minutes, 120 minutes, 150 minutes, and 180 minutes. The t-values and p-values are calculated using an independent samples t-test to assess the significance of differences between the groups.

Heart Rate (Minutes)	Group B		Group F		t-value	P-value
	Mean	Standard Deviation	Mean	Standard Deviation		
10 min	82.9	16.945	80.87	13.88	0.508	0.613
20 min	79.77	16.931	80.90	17.285	-0.256	0.798
30 min	76.47	16.150	78.23	77.33	-0.122	0.684
60 min	74.40	14.426	77.33	15.519	-0.757	0.451
90 min	75.63	16.331	77.33	13.135	-0.444	0.699
120 min	80.37	16.391	82.38	14.603	-0.502	0.733
150 min	75.00	13.191	84.67	12.644	-2.899	0.224
180 min	105.00	10.698	86.00	6.00	8.484	0.111

No significant difference was obtained in HR at any point in time in both groups (P > 0.05). Intraoperative SBP was measured in both groups, as shown in Table 3.

**Table 3:** Comparing intraoperative diastolic blood pressure (DBP) F: fractionated dose; B: bolus dose; NIDBP: non-invasive diastolic blood pressure NIDBP was recorded at 10 minutes, 20 minutes, 30 minutes, 60 minutes, 90 minutes, 120 minutes, 150 minutes, and 180 minutes The t-values and p-values are calculated using an independent samples t-test to assess the significance of differences between the groups.

NIDBP (at time interval)	Group B		Group F		t-value	p-value
	Mean	Standard Deviation	Mean	Standard Deviation		
NIDBP 10	81.52	10.894	81.57	12.136	-0.017	0.987
NIDBP 20	70.70	15.381	71.53	14.597	-0.214	0.830
NIDBP 30	70.17	14.367	68.97	9.870	0.377	0.707
NIDBP 60	68.97	10.746	69.67	8.980	-0.274	0.785
NIDBP 90	69.04	8.533	74.90	8.899	-2.603	0.025
NIDBP 120	74.38	6.859	73.92	10.226	0.205	0.888
NIDBP 150	77.17	10.704	79.17	10.477	-0.731	0.750
NIDBP 180	72.00	9.689	76.33	13.650	-1.417	0.809

A statistically significant increase in DBP was observed in Group F (74.90 mmHg) compared to Group B (69.04 mmHg) at the 90-minute (p = 0.025), indicating better hemodynamic stability at that time interval with fractionated dosing. No significant differences were noted at other time points (p > 0.05). Intraoperative MAP was measured in both groups, as shown in Table 4.

**Table 4:** Comparing intraoperative mean arterial pressure (MAP) F: fractionated dose, B: bolus dose, MAP was recorded at 10 minutes, 20 minutes, 30 minutes, 60 minutes, 90 minutes, 120 minutes, 150 minutes, and 180 minutes The t-values and p-values are calculated using an independent samples t-test to assess the significance of differences between the groups.

MAP (at time interval)	Group B		Group F		t-value	p-value
	Mean	Standard Deviation	Mean	Standard Deviation		
MAP 10	100.53	13.077	99.53	12.752	0.300	0.765
MAP 20	86.10	16.344	87.33	14.838	-0.305	0.761
MAP 30	85.80	17.285	85.57	10.909	0.062	0.950
MAP 60	84.27	12.253	85.40	8.775	-0.411	0.682
MAP 90	84.63	9.985	90.71	10.174	-2.336	0.043
MAP 120	89.75	6.875	90.46	11.587	-0.289	0.839
MAP 150	93.50	11.996	97.00	11.730	-1.143	0.620
MAP 180	81.00	12.265	94.00	15.524	-3.599	0.544

At 90 minutes, Group F demonstrated a significantly higher MAP (90.71 mmHg) compared to Group B (84.63 mmHg) (p = 0.043), suggesting better maintenance of hemodynamic stability with fractionated dosing at that time interval. No significant differences in MAP were observed at other time points (p > 0.05).

Also, on comparing the number of times vasopressors were used in both groups, it was found that vasopressors were used more in Group B (1.30 times) as compared to Group F (0.67 times) (p=0.042). In the present study, in the bolus group, 23 (76%) patients required a vasopressor, whereas in the fractionated dose group, only 12 (40%) required a vasopressor agent. The distribution was highly significant with a p-value of 0.001.

## Discussion

SA is the most common mode of regional anesthesia for lower limb surgeries. The onset of action of SA is rapid, and dense neuronal blockade is achieved. The common side effect of SA is deterioration in hemodynamic parameters like blood pressure, HR, MAP, etc. Patients of the geriatric age group have poor cardiopulmonary reserves and show exaggerated derangements in hemodynamic profile under SA. Giving a total dose of local anesthetic in two fractions with a time gap maintains the advantage of SA with the added benefit of fewer derangements in the above-stated parameters. The duration of analgesia is longer, and the vasopressor requirement is less when fractionated dosing is done.

Fractionated dosing of SA is when a local anesthetic is injected into the subarachnoid space in fractions with a time gap. It not only reduces the degree of hypotension but also provides prolonged duration of action and postoperative analgesia. Sometimes, a bolus dose of the local anesthetic agent in SA causes more hypotension [16]. This study compared the effect of injectable local anesthetic, i.e., heavy bupivacaine 0.5%, in the subarachnoid space by giving the drug in two different methods in elderly patients undergoing lower limb surgeries.

In our study, the fractionated group and the bolus group received a mean dose of 2.73 ml, which was comparable between the two groups (p=1.00). Similarly, Badheka et al. compared the bolus group and the fractionated group and noted that the bolus group received a mean (range) dose of 2.14 (2-2.4) ml, whereas the fractionated group received a mean (range) dose of 2.2 (2-2.6) ml, which were comparable between the two groups. It was similar to our study as well [16]. Harten et al. also observed that bolus and fractionated doses were comparable in their study [12]. Patel et al. compared the effect of two dosage regimens, bolus dose (2.15 ± 0.05 ml) versus fractionated dose (2.16 ± 0.04 ml), and they also noted both doses were comparable [17].

In our study, when we analyzed the hemodynamic variables like HR, SBP, DBP, and MAP, a statistically significant difference was obtained only at 90 min., where DBP was higher in Group F (74.90 mmHg) as compared to Group B (69.04 mmHg) (p=0.025). Similarly, at 90 min, MAP was higher in Group F (90.71 mmHg) as compared to Group B (84.63 mmHg) (p=0.043). However, no other statistical difference was found at any other time interval.

Badheka et al. concluded that fractionated doses provide better hemodynamic stability; they found higher MAP and HR at 5 min, 10 min, 15 min, 30 min, 45 min, and 60 min in patients receiving fractionated dosing of heavy bupivacaine [16]. On the contrary, in our study, the difference between the mean heart rates of the two groups was not significant. In our study, the finding of MAP was statistically significant in Group F as compared to Group B at 90 min. Many factors may account for this difference in our study. Firstly, because of the limited sample size, the power to detect smaller differences at other time points may have been reduced. Secondly, in elderly patients, intraoperative hemodynamic parameters are influenced by multiple dynamic variables, such as fluid shifts, spread of anesthesia, and surgical stimuli, which can contribute to variability in readings over time.

The study by Favarel et al. was done on patients posted for surgery of hip fracture, which concluded that intraoperative vitals were more stable in patients who received a titrated dose of bupivacaine than those who received SDSA, which was similar to our study at some point [3]. Patel et al. observed that patients were hemodynamically more stable in Group F as compared to Group B [17]. In Khare and Nema’s study, patients of Group F showed a better hemodynamic profile as compared to patients of Group B, where the MAP was higher in Group F than in Group B at 0 min, 5 min, 10 min, 15 min, 30 min, 45 min, and 60 min. [11].

In our study on comparing the number of times vasopressors were used in both groups, it was found that vasopressors were used significantly less in Group F (0.67 times) as compared to Group B (1.3 times) (p=0.042). It was also seen that in the bolus group, 23 (76%) patients required vasopressors, whereas in the fractioned dose group, only 12 (40%) required vasopressor agents. The distribution was highly significant with a p-value of 0.042. Similarly, Badheka JP et al. found that the requirement of vasopressors was lower in Group F, as it was required in five patients (16.66%) as compared to bolus dosage, where it was required in 14 patients (46.66%). The findings of our study were similar to this [16].

Patel et al. studied a randomized trial in 60 patients to compare the effect fractionated dose and a bolus dose and observed that four patients (13.33%) required vasopressors in Group F and 11 patients (36.66%) required vasopressors in Group B, where P = 0.03 [17]. In another study by Khare and Nema, when a single bolus dose of 0.5% heavy bupivacaine was used, the degree of hypotension was higher. However, the fractionated dose was associated with a minimal requirement of vasopressors (13.33%) as compared to the bolus dose (43.33%) (P = 0.013), which was consistent with our study as well [11].

Limitation

This study aimed to evaluate whether fractionated spinal dosing offers better hemodynamic stability compared to bolus dosing in elderly patients. Although statistically significant differences in MAP and DBP were found at the 90-minute interval, no consistent differences were observed across all time intervals. These isolated findings in MAP and DBP, though suggestive, do not offer sufficient evidence to draw firm clinical conclusions, especially given the limited sample size and short observation period.

The reduced vasopressor requirement in the fractionated group in the current study is noteworthy, supporting the hypothesis that slower or staged dosing may attenuate the extent of sympathetic blockade. However, factors such as intraoperative fluid shifts, surgical stimulation, patient comorbidities, and baseline autonomic tone may have influenced the observed outcomes.

Also, while our calculated sample size was based on standard assumptions (α = 0.05, power = 80%, effect size derived from prior literature), practical constraints limited the final cohort to 30 patients per group. This may have reduced the study's power to detect meaningful differences across multiple time points.

## Conclusions

In current study, fractionated dose of hyperbaric bupivacaine showed better intraoperative hemodynamic stability at isolated time interval when compared to bolus dose in elderly patients undergoing lower limb surgeries. Also, the reduced vasopressor requirement in fractionated group is statistically significant and promising, the short follow-up and methodological limitations require cautious interpretation. Larger, multicenter trials and longer follow-up are essential to determine the clinical utility of fractionated dosing in this population.
